# Therapeutic Outcomes of High Dose-Dexamethasone versus Prednisolone + Azathioprine, Rituximab, Eltrombopag, and Romiplostim Strategies in Persistent, Chronic, Refractory, and Relapsed Immune Thrombocytopenia Patients

**DOI:** 10.3390/ph16091215

**Published:** 2023-08-29

**Authors:** Eman Mostafa Hamed, Ahmed R. N. Ibrahim, Mohamed Hussein Meabed, Ahmed M. Khalaf, Doaa Mohamed El Demerdash, Marwa O. Elgendy, Haitham Saeed, Heba F. Salem, Hoda Rabea

**Affiliations:** 1 Department of Clinical Pharmacy, Faculty of Pharmacy, Nahda University (NUB), Beni-Suef 62521, Egypt; marwa.elgendy@nub.edu.eg; 2 Department of Clinical Pharmacy, College of Pharmacy, King Khalid University, Abha 61421, Saudi Arabia; 3 Department of Pediatrics, Faculty of Medicine, Beni-Suef University, Beni-Suef 62521, Egypt; m1hmeabed2@med.bsu.edu.eg; 4 Department of Internal Medicine and Clinical Hematology, Beni-Suef University, Beni-Suef 62521, Egypt; dr.ahmed201176@yahoo.com; 5 Department of Internal Medicine and Clinical Hematology, Faculty of Medicine, Cairo University, Giza 54212, Egypt; dr_eldemerdash@kasralainy.edu.eg; 6 Department of Clinical Pharmacy, Faculty of Medicine, Beni-Suef University Hospitals, Beni-Suef University, Beni-Suef 62521, Egypt; 7 Clinical Pharmacy Department, Faculty of Pharmacy, Beni-Suef University, Beni-Suef 62521, Egypt; haitham.sd1@gmail.com (H.S.); hoda.ahmed@pharm.bsu.edu.eg (H.R.); 8 Department of Pharmaceutics and Industrial Pharmacy, Faculty of Pharmacy, Beni-Suef University, Beni-Suef 62521, Egypt; heba.salem@nub.edu.eg; 9 Pharmaceutics and Industrial Pharmacy Department, 6 October Technological University, Giza 62521, Egypt

**Keywords:** autoimmune disease, relapsed, chronic, refractory immune thrombocytopenia, dexamethasone, prednisolone, azathioprine, rituximab, eltrombopag, romiplostim

## Abstract

Background: Primary immune thrombocytopenia (ITP) is an inflammatory autoimmune disease that can be managed with several treatment options. However, there is a lack of comparative data on the efficacy of these options in different phases of the disease. Aim of the study: This study aimed to evaluate the efficacy of high-dose Dexamethasone (HD-DXM), Prednisolone + Azathioprine, Rituximab, Eltrombopag, and Romiplostim schedules in persistent, chronic refractory or relapsed Egyptian ITP patients with a platelet count ≤30 × 10^9^/L. The primary outcome measure was a sustained increase in platelet counts over 50 × 10^9^/L for an additional 12 months without additional ITP regimens. The study also aimed to identify a suitable treatment regimen with a long remission duration for each phase of ITP. Results: Prednisolone + Azathioprine was significantly more effective in achieving an overall response in persistent patients than Romiplostim, high-dose Dexamethasone, and Rituximab. (90.9% vs. 66.6, [Odds ratio, OR: 5; confidence interval, CI 95% (0.866–28.86)], 45%, [OR: 0.082, CI 95% (0.015–0.448)] and, 25%, [OR: 30, CI 95% (4.24–211.8)], respectively, *p*-value < 0.01). Eltrombopag was significantly more effective in achieving a durable response in refractory ITP than HD-DXM, Rituximab, and Prednisolone; (80% compared to 32.2% [OR: 0.119, CI 95% (0.035–0.410)], 22.2% [OR:0.071, CI 95% (0.011–0.455)], and 18.1% [OR: 0.056, CI 95% (0.009–0.342)], respectively, *p*-value < 0.01). Conclusions: Finally, Eltrombopag following HD-DXM showed the highest percentage of patients with complete treatment-free survival times of at least 330 days. These findings could help clinicians choose the most appropriate treatment for their patients with ITP based on the phase of the disease. This trial is registered in clinicaltrials.gov with registration number NCT05861297.

## 1. Introduction

Primary immune thrombocytopenia (ITP) is an autoimmune hemorrhage disorder characterized by impairment of platelet production and immunological abnormalities resulting in platelet destruction [1]. Severe internal bleeding can occur as a consequence [2,3]. ITP affects approximately 1 in every 20,000 individuals, with females being more affected than males [4,5,6]. ITP is characterized by self-antigen-induced chronic immune system activation, ultimately leading to tissue inflammation in genetically susceptible individuals. Moreover, inflammation can cause ITP. For instance, serum uric acid, an inflammatory mediator, contributed to the pathophysiology of ITP [7]. Platelets mediate inflammation and immune-mediated disorders by releasing pro-inflammatory mediators, surface inflammation-related molecules, and interaction with endothelial cells and leukocytes [8]. Platelets modulated monocyte survival surface molecules following phagocytosed by the system of mononuclear phagocytes [9].

ITP is classified into phases based on symptoms’ duration, treatment response, and relapse after remission. Acute ITP represents the confirmed diagnosed phase, lasting about three months [10]. Some patients with ITP persist despite early treatment and progress to persistent ITP, lasting between 3 and 12 months. Chronic ITP is defined as the presence of ITP symptoms for over one year [11]. The American Society of Hematology guidelines identified a group of patients as having refractory ITP if they matched two criteria: (1) they failed splenectomy and (2) they remained to have serious ITP or a bleeding risk necessitating therapy [1,12]. Recent studies also defined the refractory term as a lack of response to one or more conventional therapies (including rituximab and thrombopoietin Receptor Agonists [13,14,15]. Relapsed ITP was described in the patients who represented recurrent thrombocytopenia after normalization of platelets while patients were both on and off-label treatment, with the greatest response to that line recorded before relapse and subsequent requiring re-therapy [16]. Corticosteroids, particularly high-dose Dexamethasone (HD-DXM) or Prednisolone (PSL), are commonly recommended for newly diagnosed ITP [17]. However, despite an initial response in 60–70% of patients, most responders experience relapse, with only 15–40% achieving a durable sustained response [18]. Second-line treatment options such as Rituximab (RTX), Azathioprine (AZA), and TPO-RAs like Eltrombopag (ELTRO) or Romiplostim (ROMP) are recommended by the American Society of Hematology guidelines for treating corticosteroid-resistant or intolerant ITP [3,17,19]. Nowadays, splenectomy is delayed and only considered following TPO-RAs and/or rituximab failure. According to the most recent American Society of Hematology (ASH) guidelines and an updated international consensus report, splenectomy is a third-line treatment option [20,21]. However, comparative data on the optimal therapeutic regimens for each phase of primary ITP are lacking.

Corticosteroids primarily exert their impact on immune thrombocytopenic purpura (ITP) through two mechanisms: (i) suppression of the reticuloendothelial system’s ability to destroy platelets that are attached to antibodies and (ii) inhibition of the generation of anti-platelet antibodies and (iii) prevention damage of megakaryocytes by macrophages in the reticuloendothelial system, such as spleen, or cytotoxic T cells in the bone marrow [22,23].

Eltrombopag increases platelet production by stimulating the proliferation and differentiation of megakaryocytes in the bone marrow via the Janus kinase/signal transducer and activator of the transcription (JAK/STAT) signaling pathway [24,25]. Romiplostim is a peptide-antibody fusion composed of two dimerized peptides conjugated to the heavy chain of IgG1 [26]. These peptides contain a 14-amino acid sequence distinct from the endogenous thrombopoietin sequence. This reduces the theoretical risk of subsequent autoantibody formation and immunogenicity, greatly reducing the concern that plagued first-generation TPO mimetics [27]. Rituximab is a monoclonal antibody that has a human-mouse chimeric structure. It specifically targets CD20 antigens present in B cells, hence exerting its therapeutic effect by reducing anti-platelet antibodies by suppressing B cell activity [28].

This study aimed to evaluate the efficacy of frontline Dexamethasone and the most commonly used second-line treatment modalities in each phase of confirmed diagnosed ITP. The study hypothesized that Eltrombopag and Romiplostim would increase the durable response compared to standard therapy in each phase of confirmed diagnosed ITP. The primary objective was to compare the effectiveness of Eltrombopag and Romiplostim with the current standard of care for treating different phases of ITP in terms of the percentage of patients who achieve a greater platelet response. The study seeks to determine the most suitable treatment regimen for each phase of primary ITP and contribute to the understanding and improvement of treatment strategies.

## 2. Results

### 2.1. Demographic Data

**Figure 1:**
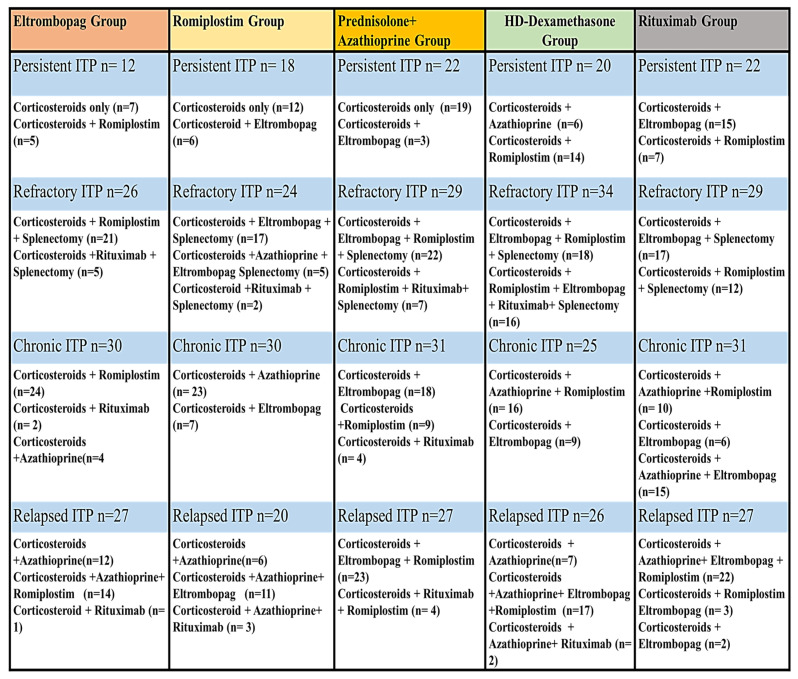
The previous treatments of patients in each ITP Phase.

Egyptian ITP patients who fulfilled the inclusion criteria were enrolled at a hematology outpatient clinic and assigned to one of five treatment groups. The patients were enrolled in outpatient hematology clinics affiliated with the Hematology department (AL-Kasr El-Einiy, health insurance hospital, and Beni-Suef University Hospital) during the period (from May 2020 to June 2023). Patients with thrombocytopenia associated with specific conditions such as lupus, chemical-induced causes, lymphoproliferative diseases, immune thyroid diseases, and chronic infections including HIV, HCV, and Helicobacter pylori were excluded from the study. Patients with liver, cardiac, or renal impairment and those who had used NSAIDs or anti-platelet medications within one month were also excluded from the study. During the study enrollment, 37 ITP patients withdrew due to non-compliance, and four were lost to follow-up. Moreover, two patients died during the study due to severe bleeding. Four hundred sixty-five patients completed the prospective study and were followed for one year and a half. Baseline clinical, hematological markers, and demographic characteristics were comparable and similar among all patients, as shown in Table 1. Moreover, the previous treatments of patients were described in Figure 1. There were no statistically significant differences in baseline data, such as weight, gender, or age concerning platelet count, among the studied groups (*p*-value > 0.05). 

### 2.2. Effect of Gender and Age on PLT Counts after Therapeutic Regimens

A statistically significant correlation was observed between gender and platelet count after therapeutic regimens in the ELTRO regimen, ROMP regimen, PSL + AZA regimen, and HD-DXM group (*p*-value = 0.001, 0.009, 0.001, and 0.037, respectively). However, no statistically significant association between gender and platelet count following RTX was found (*p*-value = 0.446). Age and platelet count were not statistically correlated following the ELTRO regimen (*p*-value = 0.141). However, a statistically significant correlation between age and platelet count was observed after therapeutic regimens in each group, including the HD-DXM regimen, PSL + AZA regimen, RTX, and ROMP group (*p*-value = 0.001, 0.001, 0.022, and 0.002, respectively). These correlations indicated that patients who received HD-DXM or PSL + AZA in the age range of 35–45 years and 45–65 years showed greater efficacy than those aged 18–35 (*p*-value < 0.001). In contrast, patients who received RTX in the age range of 18–35 years exhibited higher efficacy than those aged 35–45 years and 45–65 years (*p*-value = 0.009 and 0.006). Additionally, the ROMP regimen showed the highest elevation in platelet count among patients aged 35–45 years compared to those aged 18–35 and 45–65 years (*p*-value = 0.025 and 0.004). 

### 2.3. Effect of ITP Phases on PLT Counts after Therapeutic Regimens

A statistically significant correlation was found between ITP phases (refractory and persistent) and platelet count after the ELTRO regimen (*p*-value = 0.001), indicating that ELTRO significantly increased platelet count in refractory ITP patients compared to persistent ITP patients. Conversely, a statistically significant correlation was observed between ITP phases (persistent and refractory) and platelet count following PSL+ AZA therapy (*p*-value = 0.001), suggesting that PSL + AZA significantly increased platelet count in persistent ITP patients compared to refractory ITP patients. HD-DXM significantly elevated platelet count in relapsed patients more than in chronic ITP patients (*p*-value = 0.02). ROMP significantly increased platelet count in refractory ITP patients compared to relapsed ITP patients (*p*-value = 0.013). Lastly, the RTX group showed greater efficacy in relapsed ITP patients than in refractory ITP patients (*p*-value = 0.001). 

### 2.4. Response Results 

The overall response in persistent ITP patients who received PSL + AZA was significantly higher than those in the ROMP, HD-DXM, and RTX groups (90.9% vs. 66.6, [OR: 5, CI 95% (0.866–28.86)], 45%, [OR: 0.082, CI 95% (0.015–0.448)], and 25%, [OR: 30, CI 95% (4.24–211.8)], respectively, *p*-value < 0.01). There was no significant statistical difference between PSL + AZA and ELTRO [OR: 3.33, CI 95% (0.472–23.53)], *p*-value = 0.11). Moreover, ELTRO and ROMP significantly increased the overall response rate more than RTX among persistent ITP patients (75% and 66.6% vs. 25%, [OR: 0.11, CI 95% (0.018–0.705)], and [OR: 0.167, CI 95% (0.033–0.853)], *p*-value < 0.01). A statistically significant difference was highlighted in the prevalence of sustained response (SR) in persistent ITP patients who received ROMP, ELTRO, and RTX compared to PSL + AZA, with SR rates of 83.3% [OR: 0.035, CI 95% (0.005–0.249)], 66.6% [OR: 0.088, CI 95% (0.014–0.562)], and 66.6% [OR: 0.088, CI 95% (0.006–1.308)], vs. 15% (*p*-value < 0.01). A statistically significant difference was also depicted in the SR in persistent ITP patients who received ROMP, ELTRO, and RTX compared to HD-DXM, 83.3% [OR: 0.025, CI 95% (0.002–0.328)], 66.6% [OR: 0.063, CI 95% (0.005–0.760)], and 66.6% [OR: 0.063, CI 95% (0.003–1.496)], vs. 11.1%, *p*-value < 0.01). As a result, the HD-DXM and PSL showed the highest significant relapsed patients as compared to RTX, ELTRO, and ROMP among persistent ITP patients (88.8%, 85% vs. 33.3%, 33.3%, and 16.6%, respectively, *p*-value < 0.01).

Among chronic ITP patients, ELTRO and ROMP achieved a significantly higher overall response rate than RTX (96.6% [OR: 0.063, CI 95% (0.007–0.587)], 86.6% [OR: 0.282, CI 95% (0.066–1.20)], vs. 64.7%, respectively (*p*-value < 0.01). Additionally, a statistically significant difference was found in the incidence of overall response rate in the ELTRO group compared to HD-DXM in chronic patients (96.6% vs. 68% [OR: 0.073, CI 95% (0.008–0.638)], respectively, *p*-value = 0.004). ELTRO and HD-DXM sustained the response significantly more than PSL + AZA among chronic patients (62% and 41.1% vs. 23%, [OR: 0.183, CI 95% (0.056–0.597)] and [OR:0.233, CI 95% (0.618–8.810)], respectively, *p*-value < 0.01). Moreover, ELTRO and HD-DXM also sustained the response significantly more than RTX among chronic patients (62% [OR: 0.136, CI 95% (0.025–0.748)] and 41.1% [OR: 3.150, CI 95% (0.515–19.271)] vs. 18.1%, respectively, *p*-value < 0.01). Therefore, RTX and PSL resulted in significantly higher relapsed patients than HD-DXM and ELTRO (81.8% and 76.9% vs. 58.8% and 37.9%, respectively; *p*-value < 0.01).

**Figure 2:**
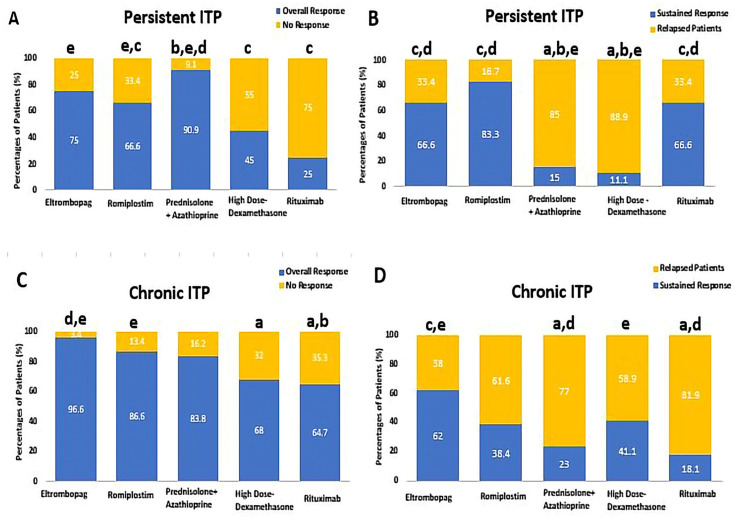
Comparison between Eltrombopag, Romiplostim, Prednisolone + Azathioprine, high-dose Dexamethasone, and Rituximab according to percentages of patients’ response, (**A**) the percentages of patients who achieved the overall response rate (ORR) and no response (NR) in persistent ITP patients, (**B**) the percentages of patients who achieved the sustained response (SR) and relapse in persistent ITP patients, (**C**) the percentages of patients who achieved the overall response rate (ORR) and no response (NR) in chronic ITP. (**D**) The percentages of patients who achieved the sustained response (SR) and relapse in chronic ITP. ^a^ Significantly different from the Eltrombopag regimen at *p*-value < 0.05. ^b^ Significantly different from the Romiplostim regimen at *p*-value < 0.05. ^c^ Significantly different from the Prednisolone + Azathioprine regimen at *p*-value < 0.05. ^d^ Significantly different from the high-dose Dexamethasone regimen at *p*-value < 0.05. ^e^ Significantly different from the Rituximab regimen at *p*-value < 0.05.

These corresponding patient responses to HD-DXM, PSL + AZA, RTX, ELTRO, and ROMP in persistent and chronic ITP patients are depicted in Figure 2 and summarized in Table 2.

Among refractory ITP patients, ELTRO, ROMP, and HD-DXM significantly increased the overall response rate compared to RTX, with response rates of 96.1% [OR: 0.051, CI 95% (0.006–0.478)], 91.6% [OR: 0.117, CI 95% (0.020–0.674)], and 91.1% [OR:8.037, CI 95% (1.71–37.59)] compared to 56.2%, respectively (*p*-value < 0.01). Additionally, ELTRO, ROMP, and HD-DXM significantly also increased the ORR more than PSL + AZA, 96.1% [OR: 0.024, CI 95% (0.003–0.207)], 91.6% [OR: 0.056, CI 95% (0.011–0.284)], and 91.1% [OR: 16.90, CI 95% (4.159–68.74)] vs. 37.9%, respectively (*p*-value < 0.01). The sustained response was significantly higher in refractory patients who received ELTRO compared to HD-DXM, RTX, and PSL + AZA (80% compared to 32.2% [OR: 0.119, CI 95% (0.035–0.410)], 22.2% [OR: 0.071, CI 95% (0.011-.0.455)], and 18.1% [OR: 0.056, CI 95% (0.009–0.342)], *p*-value < 0.01). ROMP also significantly elevated the SR in refractory patients compared to HD-DXM, RTX, and PSL + AZA (63.6% compared to 32.2% [OR: 0.272, CI 95% (0.086–0.859)], 22.2% [OR: 0.163, CI 95% (0.027–0.983)], and 18.1% [OR: 0.127, CI 95% (0.022–0.739)], *p*-value < 0.01). More patients on immunomodulators (Prednisolone + Azathioprine, Rituximab, and HD-DXM) relapsed than those on TPORAs (Romiplostim and Eltrombopag), 81.8%, 77.7%, and 67.7% vs. 36.3% and 20%, respectively; *p*-value < 0.01).

**Figure 3:**
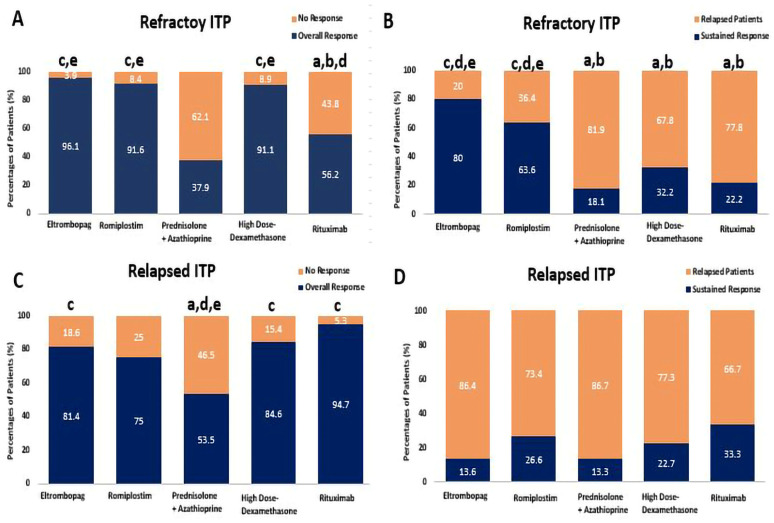
Comparison between Eltrombopag, Romiplostim, Prednisolone + Azathioprine, high-dose Dexamethasone, and Rituximab according to percentages of patients’ response, (**A**) the percentages of patients who achieved the overall response rate (ORR) and no response (NR) in refractory ITP patients, (**B**) the percentages of patients who achieved the sustained response (SR) and Relapse in refractory ITP patients, (**C**) the percentages of patients who achieved the overall response rate (ORR) and no response (NR) in relapsed ITP. (**D**) The percentages of patients who achieved the sustained response (SR) and Relapse in relapsed ITP. ^a^ Significantly different from the Eltrombopag regimen at *p*-value < 0.05. ^b^ Significantly different from the Romiplostim regimen at *p*-value < 0.05. ^c^ Significantly different from the Prednisolone + Azathioprine regimen at *p*-value < 0.05. ^d^ Significantly different from the high-dose Dexamethasone regimen at *p*-value < 0.05. ^e^ Significantly different from the Rituximab regimen at *p*-value < 0.05.

Furthermore, among relapsed ITP patients, the overall response rate was significantly higher in those who received RTX, HD-DXM, and ELTRO compared to PSL + AZA, with response rates of 94.7% [OR: 0.064, CI 95% (0.007–0.548)], 84.6% [OR: 4.76, CI 95% (1.301–17.46)], and 81.4% [OR: 0.262, CI 95% (0.077–0.890)], compared to 55.5%, respectively (*p*-value < 0.01). These corresponding patient responses to HD-DXM, PSL + AZA, RTX, ELTRO, and ROMP in refractory and relapsed ITP patients are depicted in Figure 3 and summarized in Table 2. In addition, the summary of the most effective therapeutic regimen in each ITP Phase is shown in Table 3.

The overall response percentages were calculated from the total number of patients in each group. The sustained and relapsed percentages were calculated from the patients who achieved the overall response. The complete and partial responses were calculated from the patients who achieved the overall response. * *p*-value ≤ 5% represents the comparison between five different groups.

### 2.5. Relapse-Free Survival 

**Figure 4:**
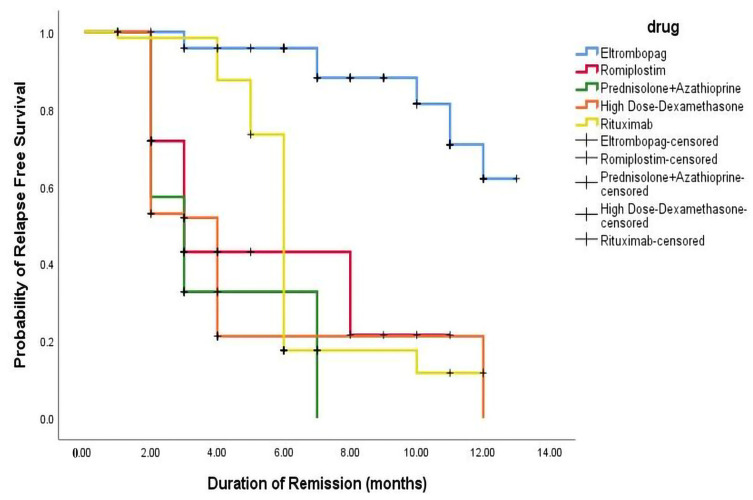
Kaplan–Meier appraisal of relapse-free survival across all regimens.

The Kaplan–Meier analysis highlighted that early CR responders had a significantly lower relapse risk than patients treated with HD-DXM (*p*-value = 0.001). The relapse-free survival rates varied among the different therapeutic regimens, emphasizing the importance of selecting the appropriate treatment based on patient characteristics and the ITP phase. The proportion of relapsed patients treated with HD-DXM who experienced relapse within 12 months was 51.7%, with 26. 7% relapsing within 2 and 4 months, respectively. In comparison, the relapse rates for patients treated with PSL + AZA were 54.2%, 28.8%, and 16.9% at 2, 3, and 7 months respectively. Among patients treated with RTX, 62.2% experienced relapse within 6 months, with 17.2% and 20.6% relapsing within 4 and 12 months, respectively. Additionally, the proportion of relapsed patients treated with ELTRO at 11, 3, and 7 months was 68.4%, 10.5%, and 21%, respectively. In comparison, 64.8% of patients treated with ROMP relapsed within 2 months, and 21.6% and 13.5% relapsed within 3 and 8 months. These findings are profiled in Figure 4. 

These findings demonstrate that the therapeutic regimens, including ELTRO, ROMP, HD-DXM, PSL + AZA, and RTX, have variable effects on PLT counts and response rates in different subgroups of ITP patients. PSL + AZA showed a higher overall response rate in persistent ITP patients, while ELTRO and ROMP exhibited higher response rates in chronic, refractory, and relapsed ITP patients. HD-DXM demonstrated lower response rates and a higher relapse rate than other regimens. During the treatment period, two fatalities occurred: one from cardiac arrest in the ELTRO group and one from intracranial hemorrhage in the PSL + AZA group. However, no deaths were believed to be directly related to the treatment.

Notably, these results are based on a specific cohort of Egyptian ITP patients and may not be directly applicable to other populations. Further studies with larger sample sizes and diverse patient populations are warranted to validate these findings and provide more comprehensive insights into the management of ITP.

## 3. Discussion

This study represents the first randomized investigation into the efficacy of different treatment regimens, including ELTRO, ROMP, PSL + AZA, HD-DXM, and RTX, in persistent, chronic, refractory, and relapsed ITP patients. The results demonstrated that all treatment-line regimens increased the platelet count throughout the one-and-a-half-year follow-up. Notably, ELTRO exhibited the highest proportion of patients (68.4%) achieving completely treatment-free survival intervals of at least 330 days.

ELTRO and ROMP showed a significantly higher overall response rate (ORR) in persistent ITP patients compared to RTX (75% and 66.6% vs. 25%, *p*-value < 0.01). These findings are closely in line with a retrospective network meta-analysis study by Puavilai et al., which also found higher efficacy of ROMP and ELTRO in persistent ITP patients compared to RTX (*p*-value < 0.001) [29]. 

In chronic ITP patients, ELTRO and ROMP achieved a significantly higher ORR than RTX (96.6% and 86.6% vs. 64.7%, *p*-value < 0.01). The mechanism behind ELTRO’s efficacy in chronic ITP was proposed to involve its binding to members of the BCL-2 family (BCL2, BAX, and BCL2L1), preventing the pro-apoptotic member BAX from mediating apoptosis [30,31]. 

Recent studies have highlighted the role of apoptosis in controlling platelet lifespan and survival in chronic ITP patients [2]. TPO-RAs have been suggested to reduce platelet apoptosis [30], and their immunomodulatory activity on monocyte/macrophage plasticity further supports the findings of this study [30]. 

One of the most important observations was that ELTRO exposed the highest percentages of SR (62%) in chronic ITP patients and 80% in refractory patients. Another study confirmed that ELTRO has immunomodulatory effects on the B cell lym-phoma-2 (Bcl-2) family. The Bcl-2 family are critical regulators of the process of apoptosis, or programmed cell death [30,31]. ELTRO’s immunomodulatory effects and impact on megakaryocytes likely contribute to the elevation in platelet mass. Based on the results of recent works, using ELTRO may be beneficial in chronic and refractory ITP patients [31].

HD-DXM showed a higher overall response rate in chronic ITP patients than in persistent ITP patients, likely due to its role in reducing apoptosis and increasing PLT counts [2]. Although the overall response to RTX was relatively high (64.7% and 56.2%), the sustained response was not encouraging in chronic and refractory ITP patients (18.1% and 22.2%). This higher relapse rate aligns with a multi-center study by Ayat et al., which reported a 15% sustained response rate to RTX [5]. The hypothesis of increased relapse in chronic or refractory ITP is attributed to the B-cell rebound effect caused by RTX [32].

Among refractory ITP patients, ELTRO, ROMP, and HD-DXM significantly increased the ORR compared to RTX and PSL + AZA (96.1%, 91.6%, and 91.1% vs. 56.2% and 37.9%, respectively, *p*-value < 0.01). These findings align with a study by Patrizio Mazza et al., which found significantly higher ORR for ELTRO and ROMP compared to RTX (94.2% and 80% vs. 24.1%) in refractory ITP patients [32]. Additionally, the sustained response was significantly higher in refractory patients who received ELTRO and ROMP compared to HD-DXM, RTX, and PSL+ AZA (80% and 63.6% vs. 32.2%, 22.2%, and 18.1%, *p*-value < 0.01). Recent studies have highlighted the ability of ELTRO to reverse the macrophage (M1)-associated characteristics of ITP [30], and TPO-RAs have been shown to have additional immunomodulatory activity, including the restoration of monocyte dynamics and the balancing of T-helper cell types [30].

These factors contribute to increased PLT survival and sustained platelet response, explaining the higher ORR observed with HD-DXM (91.1%) in refractory ITP patients. Another recent study recommended HD-DXM as an effective regimen in refractory ITP patients due to its correction of macrophage (M1/M2) polarization imbalance in the spleen, particularly in patients who have undergone splenectomy [33,34]. 

One of the most severe and life-threatening complications of severe ITP is intracranial hemorrhage (ICH). The study reports the case of a 49-year-old male patient with ICH who received PSL + AZA and ultimately withdrew from the study, succumbing to the condition despite rescue treatment. This unfortunate event is consistent with a previous study that reported similar occurrences [35]. It should be noted that the patient’s death could not be attributed solely to cardiac damage, as the patient also had deep vein thrombosis. Similarly, another study showed sudden cardiac death in a 64-year-old man who received ELTRO [36].

The RTX group demonstrated greater efficacy in relapsed ITP patients than in chronic ITP patients (94.7% vs. 64.7%, *p*-value = 0.034). These results are higher than those reported by Huyen Tran et al., who found an ORR to RTX of 59% in relapsed ITP patients compared to 38% in chronic ITP patients (*p*-value < 0.01) [37]. 

TPO-RAs had an encouraging impact on this study. The efficacy of TPO-RA in the first-line setting is not supported by sufficient evidence, according to recent studies [3,38,39,40]. These studies demonstrated that combining TPO-RAs and Dexamethasone is a potential first-line treatment for ITP. In contrast, several studies have been published supporting TPO-RAs therapy’s efficacy in the second-line setting [41,42,43].

Tjønnfjord et al. recommended initiating RTX treatment in relapsed ITP patients instead of resorting to splenectomy [44]. HD-DXM showed the highest efficacy in relapsed ITP, with 84.6% of patients experiencing a noticeable response and a mean platelet count of 123 × 109/L. This rapid response can be an effective salvage therapy for individuals with relapsed ITP experiencing clinical bleeding as a cost-effective alternative to IVIG or anti-Rh (D). ELTRO-treated patients were more likely to achieve a treatment-free interval of at least 330 days compared to ROMP-treated patients, corroborating findings from a retrospective study conducted over a nine-year follow-up period [45]. 

However, high-dose Dexamethasone and Rituximab showed high initial response rates, and the sustainability of their responses over a long duration was challenging. Eltrombopag and Romiplostim yielded sustained platelet count responses in chronic and refractory immune thrombocytopenia patients. In comparison, patients who received high-dose Dexamethasone, Prednisolone + Azathioprine, and Rituximab experienced higher relapse rates. This study offered the most suitable regimen in all phases of immune thrombocytopenia, even in persistent, chronic, refractory, and relapsed patients.

We highlight several advantages of our study, including the randomized, controlled design, multiple outcome measures, multiple regimen comparisons, and the inclusion of a long-term follow-up period. Extensive primary Pharmaco-epidemiological studies are required to compare the clinical outcomes of the three immune modulators and the two TPO-RAs in each ITP phase. We also acknowledge some limitations of our study, including the fact that the patients with secondary thrombocytopenia were not included and the lack of blinding among the treatment providers. 

## 4. Materials and Methods 

### 4.1. Patients Selection

This study recruited 467 Egyptian primary immune thrombocytopenia patients with bleeding from the outpatient hematology clinics in three centers affiliated with the Hematology department (AL-Kasr El-Einiy, Health Insurance Hospital, and Beni-Suef University Hospital). Patients aged 18 years and older with a primary diagnosis of severe ITP were eligible for enrollment. The Research ethical committee of the pharmacy faculty at Beni-Suef University provided approval (REC-H-PhBSU-22016), and each patient signed an informed consent form before participating in the randomized study. The inclusion criteria were confirmed diagnosed ITP patients with a baseline peripheral platelet count (PLT) < 30 × 10^9^/L, an average age of 18–65 years, and normal liver, cardiac, and kidney function. The exclusion criteria were patients with a secondary ITP diagnosis, life-threatening bleeding, and hepatic, pulmonary, cardiac, or renal problems, as well as patients who had received anti-platelets or non-steroidal anti-inflammatory drugs (NSAIDs) within a month of study initiation and those with a history of cancers, osteoporosis, or diabetic mellitus. PLT counts were measured monthly to ensure the regimen’s efficacy on the patients.

Moreover, we described the persistent ITP patients who had ITP that lasted between 3 and 12 months after diagnosis, While the chronic ITP patients had ITP lasting for more than 12 months [11]. Moreover, we described the refractory ITP who relapsed after splenectomy and initial response to conventional therapies (including rituximab and thrombopoietin Receptor Agonists [13,14,15]. The relapsed ITP patients who relapsed after normalization of platelets while patients were both on and off-label treatment, with the greatest response to that line recorded before relapse and subsequent requiring re-therapy [16,46]. 

### 4.2. Study Design

This controlled multi-center prospective randomized study recruited 467 patients (370 females) with primary/persistent, chronic, refractory, or relapsed ITP. Primary ITP was diagnosed by excluding potential causes of isolated thrombocytopenia, including malignancy, lupus, Helicobacter pylori infection, human immunodeficiency virus (HIV), hepatitis C virus (HCV), and drug-induced. A stratified randomization method was applied with closed envelopes involving “Name of the intervention” labels. Eligible patients were asked to select one of these envelopes to allocate to one of the five groups. All patients were initiated with frontline corticosteroids (high-dose Dexamethasone) as a first-line approach treatment for ITP with a dose of 40 mg daily for four days immediately in a 28-day cycle after the diagnosis of ITP [17,47]. 

#### 4.2.1. Interventions

The enrolled patients who met the inclusion criteria were randomly assigned to one of the five groups. Group I received a daily oral dose of 50 mg of Eltrombopag, to be taken four hours before or after meals for six months [48]. Group II patients received a weekly subcutaneous injection of Romiplostim at a dose of 3 μg/kg (ranging from 1 to 10 μg/kg) for six months [29]. Group III patients received 20 mg of Prednisolone three times daily for two weeks and 100 mg of oral Azathioprine once daily. The Prednisolone dose was then tapered throughout the following weeks (six weeks, including treatment and taper) till discontinuation while continuing with Azathioprine 100 mg for six months [5,17]. Group IV (control group) received an IV pulse of HD-DXM therapy with 40 mg daily for four days in a 28-day cycle. The cycle was repeated once each month to complete the six cycles [49]. Group V patients received 375 mg/m^2^ (500 mg) of intravenous injection of RTX once weekly for one month [50]. 

The outcome measures were evaluated at baseline, at the end of therapy, and after a 12-month free treatment period. All therapeutic regimen doses were adjusted according to the platelet count response (Table 4). 

#### 4.2.2. Randomization

**Figure 5:**
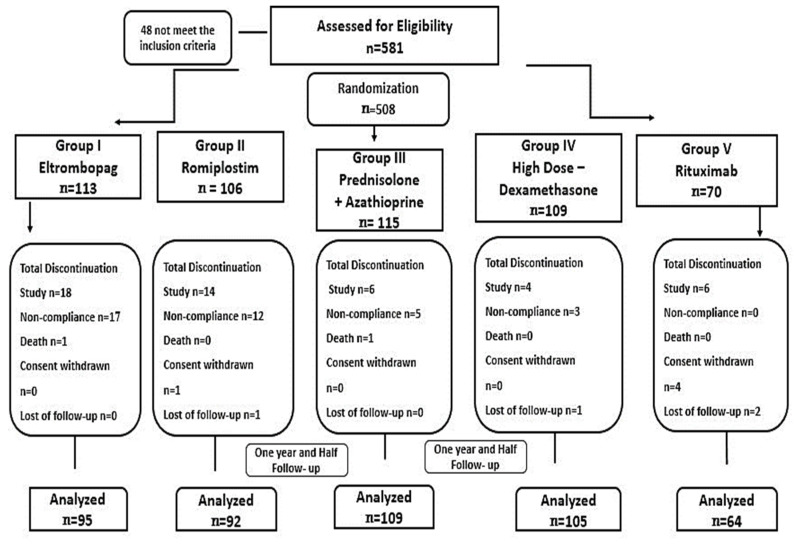
The study flow and randomization.

Four hundred sixty-seven patients with confirmed diagnosed ITP were randomly assigned to one of the five treatment groups using a closed-envelope randomization method. Stratified randomization was achieved during patients’ collection under the inclusion and exclusion criteria specification. The patients were selected from a pool of 581 patients who met the specific inclusion criteria. Patients who discontinued therapy earlier than six months due to switching to another regimen or non-compliance were excluded from the study (Figure 5).

### 4.3. Outcome Measures

The primary outcome measures were the total percentages of patients who achieved overall and sustained responses. The overall response rate (ORR) is the proportion of patients who exhibit a complete or partial response to treatment [53]. Sustained response (SR) was defined as maintaining PLT counts > 50 × 10^9^/L until the end of the study [54,55]. The secondary outcome measures were total percentages of patients who relapsed after a long period of response and relapse-free survival (RFS). Relapse was defined as the occurrence of any new bleeding manifestation requiring treatment, platelet levels below 30 × 10^9^/L after long-term response, initiation of a new type of therapy, or splenectomy due to low PLT counts [53]. RFS was defined as the interval between complete response and relapse [56]. SR and relapsed patients were calculated from the overall response. The main outcome measure was the proportion of patients who maintained PLT counts over 50 × 10^9^/L for an additional 12 months without requiring new ITP treatments. The outcome measures were evaluated at baseline, after six months of treatment, and after twelve months of treatment-free follow-up.

### 4.4. Statistical Analyses

Descriptive statistics were used to summarize the data stratified by treatment regimens (Eltrombopag, Romiplostim, Prednisolone + Azathioprine, high-dose Dexamethasone, and Rituximab). Chi-square analyses were used to compare categorical variables between each group and the others, while t-tests were used to compare continuous variables. Mann–Whitney U tests were used to compare non-normally distributed variables. Relapse-free survival analysis was performed using the Kaplan–Meier method. General linear correlation was used to examine the relationship between post-therapeutic regimens’ platelet counts and age, gender, or ITP phases for the five regimens in each phase of ITP. All statistical analyses were conducted using SPSS version 21.0 for Windows (SPSS, Chicago, IL, USA).

### 4.5. Sample Size

The sample size for the study was determined based on the results from previous studies [38,57,58,59], which have a power of 80–90%. In addition, the highest number of patients in previous randomized studies was 55 and 62 [60,61,62]. We used a two-sided overall significance level of 5% and adjusted the sample size from 60 to an overall size of 110 patients in each group, accounting for a 10% dropout rate. The minimum sample with acceptable power (85%) was 60 patients, therefore, 60 patients was the minimum acceptable number of patients for each group. 

### 4.6. Power of Sample Size

We used five comparisons (mean of pre-platelet count versus post-platelet count mean) concerning the laboratory data of the ITP patients detected by the authors. We obtained these data’s mean, standard deviation, and reference sample sizes. In light of our findings, the required sample size is 88 patients in each group for comparison to achieve 95% of the confidence interval. Therefore, we decided to randomize in the range of (60–110) patients per group, accounting for dropouts. To account for possible dropouts, we randomized the initial patient allocation to 110 patients per group, resulting in a power = 98% and a final number of participants not less than 60 to achieve a power = 85%. We ensured that these differences did not affect the validity of our statistical analyses by reporting the actual number of participants in each group and conducting appropriate statistical tests. We also described the randomization process in detail in our manuscript. The power of the sample size was calculated by G*Power 3.1.7.

## 5. Conclusions

The findings of this study shed light on pioneering observations and provide a suitable strategy for treating each ITP phase. Prednisolone + Azathioprine achieved a high early response in persistent ITP patients with low sustained response, but Eltrombopag exhibited the highest durable response. In addition, Rituximab is recommended as a predominant early treatment of relapsed ITP patients. Notably, Eltrombopag and Romiplostim showed emerging efficacy with high durable remission rates in refractory or chronic ITP patients. Further research and larger-scale investigations are needed to validate these findings and establish optimal treatment strategies for ITP in different populations.

## Figures and Tables

**Table 1:** Clinical, Laboratory, and Demographic Features of Patients with Primary Immune Thrombocytopenia in The Five Enrolled Groups.

Characteristics	Eltrombopag (*n* = 95)		Romiplostim (*n* = 92)		Prednisolone + Azathioprine (*n* = 109)		HD-Dexamethasone (*n* = 105)		Rituximab (*n* = 64)		*p*-Value
Age, median (range): years	34.3		32.5		27.8		29.5		34.5		0.09
	(18–65)		(18–65)		(18–65)		(18–65)		(18–65)		
Gender; *n* (%)											
Male	17 (17.8%)		30 (32.6%)		18 (17.4%)		15 (14.3%)		15 (23.4%)		0.60
Female	78 (82.1%)		62 (67.4%)		91 (83.4%)		90 (85.7%)		49 (76.5%)		0.08
Age (years)	Male	Female	Male	Female	Male	Female	Male	Female	Male	Female	
18–35 (%)	18.50%	81.50%	31.40%	68.60%	13.50%	0.455	19.40%	80.60%	22.70%	77.30%	0.455
35–45 (%)	19.50%	80.50%	37.50%	62.50%	19.50%	0.199	12.80%	87.20%	17.40%	82.60%	0.199
45–65 (%)	15.60%	84.40%	28%	72%	16.60%	0.331	10%	90%	31.60%	68.40%	0.331
Persistent ITP; *n* (%)	12 (12.6%)		18 (19.5%)		22 (20.1%)		20 (19%)		22 (20.1%)		0.769
Chronic ITP; *n* (%)	30 (31.5%)		30 (32.6%)		31 (28.4%)		25 (23.8%)		31 (28.4%)		0.227
Refractory ITP; *n* (%)	26 (27.3%)		24 (26.08%)		29 (26.6%)		34 (32.3%)		29 (26.6%)		0.805
Relapsed ITP; *n* (%)	27 (28.4%)		20 (21.7%)		27 (24.7%)		26 (24.7%)		27 (24.7%)		0.660

**Table 2:** Comparison between patient responses following high-dose Dexamethasone, Prednisolone + Azathioprine, Rituximab, Eltrombopag, and Romiplostim in each phase of Primary Immune Thrombocytopenia.

Treatment Response	Persistent ITP			Chronic ITP			Refractory ITP			Relapsed ITP		
	ORR (%)	SR (%)	NR (%)	ORR (%)	SR (%)	NR (%)	ORR (%)	SR (%)	NR (%)	ORR (%)	SR (%)	NR (%)
Eltrombopag (*n* = 95)	9/12 (75%)	6/9 (66.6%)	3/12 (25%)	29/30 (96.6%)	18/29 (62%)	1/30 (3.3%)	25/26 (96.1%)	20/25 (80%)	1/26 (3.8%)	22/27 (81.4%)	3/22 (13.6%)	5/27 (18.5%)
Romiplostim (*n* = 92)	12/18 (66.6%)	10/12 (83.3%)	6/18 (33.3%)	26/ 30 (86.6%)	10/ 26 (38.4%)	4/30 (13.3%)	22/24 (91.6%)	14/22 (63.6%)	2/24 (8.3%)	15/20 (75%)	4/15 (26.6%)	5/20 (25%)
Prednisolone + Azathioprine (*n* = 109)	20/22 (90.9%)	3/20 (15%)	2/22 (9.09%)	26/31 (83.8%)	6/26 (23.07%)	5/31 (16.1%)	11/29 (37.9%)	2/11 (18.1%)	18/29 (62.06%)	15 /27 (55.5%)	2/15 (13.3%)	12/27 (44.4%)
High-Dose Dexamethasone (*n* = 105)	9/20 (45%)	1/9 (11.1%)	11/20 (55%)	17/25 (68%)	7/17 (41.1%)	8/25 (32%)	31/34 (91.1%)	10/31 (32.2%)	3/34 (8.8%)	22/26 (84.6%)	5 /22 (22.7%)	4/26 (15.3%)
Rituximab (*n* = 64)	3/12 (25%)	2/3 (66.6%)	9/12 (75%)	11/17 (64.7%)	2/11 (18.1%)	6/17 (35.2%)	9/16 (56.2%)	2/9 (22.2%)	7/16 (43.7%)	18/19 (94.7%)	6/18 (33.3%)	1/19 (5.2%)
*p*-value	0.013 *	0.001 *	0.013 *	0.008 *	0.030 *	0.008 *	<0.0001 *	0.001 *	<0.0001 *	0.026 *	0.722	0.026 *

**Table 3:** The Most Effective Therapeutic Regimen in each ITP Phase.

ITP Phases	The Most Effective Drug, According to PLT Count	Overall Response, ORR (%)	Sustained Response, SR (%)	ORR *p*-Value	SR *p*-Value
Persistent ITP	Prednisolone	Prednisolone (90.9%)	Romiplostim (83.3%)	0.013 *	0.001 *
Chronic ITP	Prednisolone	Eltrombopag (96.6%)	Eltrombopag (62%)	0.008 *	0.030 *
Refractory ITP	Prednisolone	Eltrombopag (96.1%)	Eltrombopag (80%)	<0.0001 *	0.001 *
Relapsed ITP	Prednisolone	Rituximab	Rituximab	0.026 *	0.722

**Table 4:** Treatment algorithm with dose adjustments in immune thrombocytopenia patients. Further increases or decreases in therapeutic regimen doses were adjusted based on platelet response.

Corticosteroids (High Dose of Dexamethasone and Prednisolone [20]	
Platelet Response	Dose Adjustment
If platelet counts <30 × 10^9^/L	Corticosteroids are the standard initial therapeutic regimen for ITP adults: either Prednisolone at 1 mg/kg (to a maximum of 80 mg, even in patients weighing 80 kg) for two weeks, to a maximum of three weeks, or dexamethasone 40 mg/d for 4 days, repeated up to 3 times.
Platelet counts >50 ×10^9^/L	Prednisolone dose was tapered to discontinue it within 6 weeks (maximum 8 weeks), even if the platelet count decreased during the decline.
No response to the starting dose during two weeks.	Prednisolone was rapidly decreased over 1 week and stopped.
Eltrombopag [20,38]	
Platelet response	Dose Adjustments
If platelet counts <50 × 10^9^/L post at least two weeks of the regimen	The daily dose was elevated by 25 mg to a maximum of 75 mg.
Platelet counts were between 50–350 × 10^9^/L.	The Eltrombopag dose was not changed.
Platelet counts were between 350–400 × 10^9^/L.	In this condition, the daily dose was reduced by 25 mg and was delayed two weeks to judge the clinical effects.
Platelet counts >400	Eltrombopag was discontinued; PLT counts were monitored every three days and repeated at the previous dose.
Romiplostim [51,52]	
Platelet response	Dose Adjustments
If platelet counts <30 × 10^9^/L	Romiplostim was initiated subcutaneously at a dose of 1 μg/kg per week, with adjustments up to 10 μg/kg per week based on platelet response over 10 weeks.
Platelet counts (30–50 × 10^9^/L)	Romiplostim was administrated at a maximum dose of 10 μg/kg
Platelet counts, 50–200 × 10^9^/L	The dose was maintained (5–8 μg/kg).
Platelet counts > 400 × 10^9^/L	The Romiplostim was discontinued, and platelet count was evaluated each week. When the platelet count dropped below 200 × 10^9^/L, the weekly dose was resumed for 1 week, then 1 μg/kg reduction.
No response	If the platelet counts have not increased after four weeks of therapy (at a maximum of 10 mg/kg every week), the Romiplostim was stopped.
Rituximab	
Platelet response	Dose Adjustments
All Rituximab group received 375 mg/m^2^ of intravenous injection of RTX once weekly for one month.	

## Data Availability

The raw data is contained within the article.

## Abbreviations

(ITP) Immune thrombocytopenia, (HD-DXM) high-dose Dexamethasone, (OR) Odds ratio, (CI) Confidence interval, (PSL) Prednisolone, (TPO-RA) Thrombopoietin Receptor Agonists,(ELTRO) Eltrombopag, (AZA)Azathioprine, (RTX) Rituximab, (ROMP) Romiplostim, (ORR) Overall response rate, (SR) Sustained response, American Society of Hematology (ASH), (NSAIDs) non-steroidal anti-inflammatory drugs, (PLT) Platelet count, (HIV) Human immunodeficiency virus, (HCV) Hepatitis C virus, (RFS) Relapse-free survival, (NR) No response, (Bcl-2) B cell lym-phoma-2 family, (ICH) Intracranial hemorrhage.
